# Late local recurrence of dermatofibrosarcoma protuberans in the skin of female breast

**DOI:** 10.1186/1477-7819-8-48

**Published:** 2010-06-03

**Authors:** Dimitrios M Dragoumis, Leda-Aikaterini K Katsohi, Ioannis K Amplianitis, Aris P Tsiftsoglou

**Affiliations:** 1 St Luke's Hospital, Department of General Surgery, Breast Division, Panorama, 55 236, Thessaloniki, Greece; 2 Hippokrateio Hospital, Department of Cellular Pathology, Konstantinoupoleos 49, 546 42, Thessaloniki, Greece

## Abstract

Dermatofibrosarcoma protuberans (DFSP) of the breast is exceptionally obscure and late local recurrence of this entity on this site is even more uncommon. We describe such a case in a 48-year-old woman, who at the age of 35 had a DFSP excised from her right breast. Thirteen years later, she developed an ovoid mass in her right breast over the postsurgical scar area. Wide local excision of the tumor with generous tissue margin was performed and microscopic and immunohistochemical findings established the diagnosis of recurrent DFSP. No further treatment was administered and she remains well 18 months later, without tumor recurrence. We report an exceptionally rare case of local recurrence of DFSP in the female breast and discuss in detail the diagnostic and therapeutic implications of this pathology.

## Introduction

Dermatofibrosarcoma protuberans (DFSP) is a relatively uncommon neoplasm of the deep dermis and subcutaneous tissue with low-grade malignant potential. Although DFSP may have been reported in the medical literature as early as 1890, this entity was initially described by Darier and Ferrand in 1924 and was referred as "progressive and recurring dermatofibroma". In 1925, Hoffman delineated the clinicopathologic features of this lesion, which he officially termed "dermatofibrosarcoma protuberans" [1].

The clinical behavior of DFSP is characterized by progressive local growth and a propensity for local recurrence. DFSP most commonly appears on the trunk and the extremities and it frequently looks like a benign lesion. When it occurs over the breast it can be difficult to distinguish from a primary breast lesion. Recent findings from the medical literature indicate that the mainstay of treatment is wide local excision, although Mohs micrographic surgery (MMS) emerges as an alternative approach to the use of wide resection surgery with tumor-free margins. The recurrence rate after local excision tends to decrease, as the excision margins increase. Although wide excision of lesions located on the body and upper extremities allows satisfactory aesthetic results, the situation is quite difficult for sites, such as the breast, where large excision may lead to severe cosmetic deformities [2,3].

Our case highlights two noteworthy features. Firstly, DFSP of the breast is unusual, despite the fact that the trunk is the most common site involved. Secondly, our case describes a late local recurrence of this entity. A review of the literature reveals that only few late DFSP recurrences have been documented. In a review of 115 cases of DFSP, Taylor and Helwig cited a single recurrence after 20 years, while Swan et al observed a late recurrence of DFSP in the breast after 26 years [3]. To our knowledge, this is the second case in the medical literature regarding a late local recurrence of DFSP in the breast.

## Case Report

A 48-year-old woman became aware of a non-tender, ovoid mass in the medial upper quadrant of her right breast. Despite having been aware of this lesion for six months, she had not sought immediate medical treatment. The lump seemed to be near to the skin surface and was located in the middle of postoperative scar tissue. The scar measured 3 × 2 cm and recently had become stretched and nodular in consistency. Her family history was unremarkable, as well as her medical history, except from the fact that at the age of 35, she had a skin lesion excised from the same site of her right breast, which was reported as DFSP.

On physical examination, the patient had a well-circumscribed, reddish, mobile, soft mass, approximately 2 cm in diameter, and was detected in the 12-o'clock position above the right breast, on the scar tissue of previous breast biopsy. A clinical diagnosis of locally recurrent DFSP of the right breast was strongly suspected.

**Figure 1:**
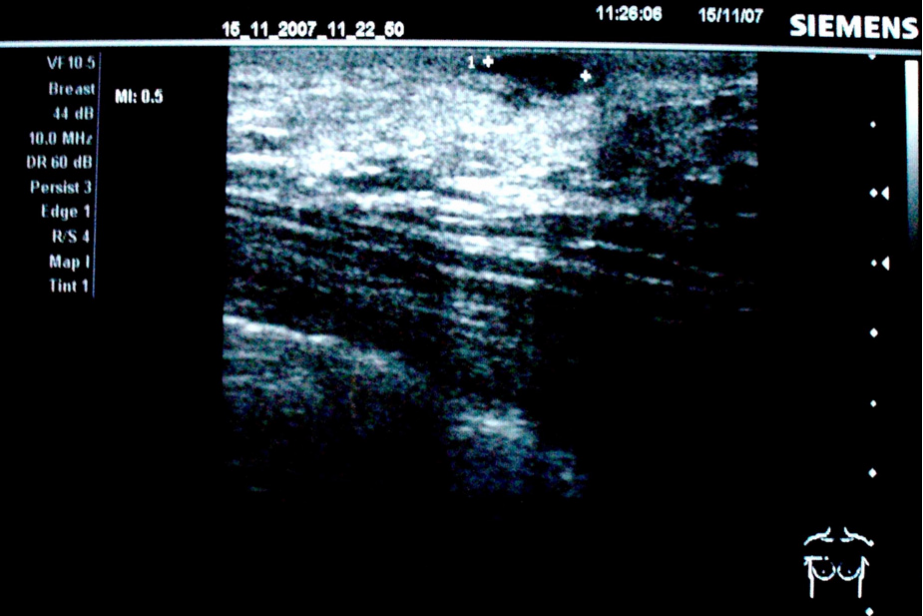
**Breast ultrasonography showing a superficial, solid, well-defined lesion with increased shadowing through transmission, simulating a benign breast neoplasm**.

Breast ultrasonography confirmed the presence of a superficial, solid, well-defined lesion on the right breast, measuring 20 mm, with increased shadowing through transmission, simulating a benign neoplasm (Figure 1). Mammography was not eventually performed because of patient's denial and lack of suspicious findings of breast parenchyma pathology on clinical and ultrasound examination. Core biopsy was then recommended and the biopsy specimen demonstrated oval to spindle-shaped cells arranged in a storiform growth pattern suggestive of DFSP. Staging investigations were subsequently performed to exclude the presence of metastatic disease. Preoperative examination consisting of a full blood count, serum kidney and liver functions, chest X-ray, as well as computed tomography (CT) of the chest, was within the normal limits.

The necessity for radical surgical treatment was thoroughly discussed with the patient, who was markedly concerned about the postoperative cosmetic result, following this second resection. The lesion was excised, including a wide 3 cm margin of normal looking skin, under local anesthesia and the defect was closed primarily.

**Figure 2:**
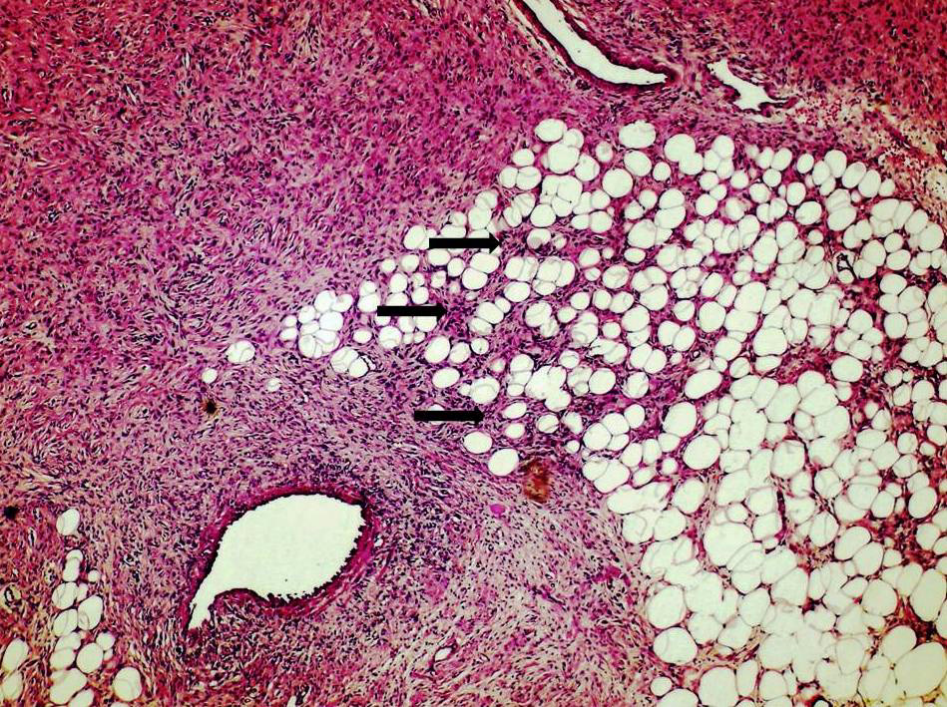
**Histologic section of the breast tumor demonstrating irregular extension of spindle-cell neoplasm (black arrows) into adipose tissue (Haematoxylin and Eosin stain; original magnification × 100)**.

**Figure 3:**
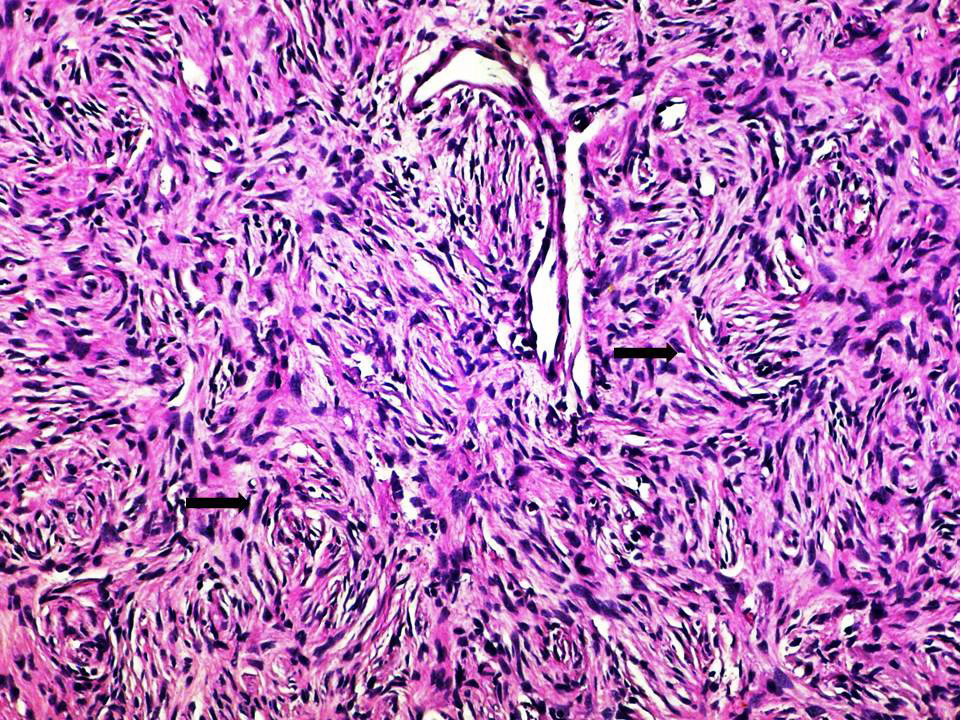
**Higher-magnification photomicrograph showing bundles of fairly uniform, spindle cells (black arrows), arranged in a prominent "storiform" or "cartwheel" pattern (Haematoxylin and Eosin stain; original magnification × 400)**.

The histological essay showed a soft tissue neoplasm comprising islands of spindle cells with plump nuclei and indistinct cytoplasm. The lesion was located in the reticular region of the dermis, causing expansion of subcuticular interlobular septa with irregular extension of spindle-cell component into adipose tissue lobule, but did not infiltrate the overlying papillary region or the epidermis (Figure 2). Microscopic sections demonstrated a tumor characterized by the presence of densely cellular uniform spindle cells arranged in whorling fascicles with a storiform pattern (Figure 3). This tumor was highly cellular, but had relatively few mitoses.

**Figure 4:**
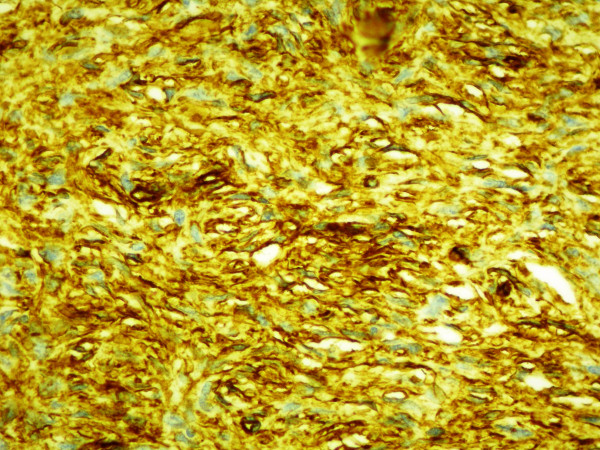
**Diffuse, strong immunohistochemical staining for CD34 of the spindle-cell component**.

The spindle cells within the dermis did not demonstrate significant staining with antibodies directed against fibrin-stabilizing factor XIII A, S-100 protein, smooth-muscle actin or cytokeratins AE1/AE3. On the other hand, the immunohistochemical profile of the tumour included intense positivity for CD34 (Figure 4). These histological and immunohistochemical findings therefore established the diagnosis of recurrent DFSP.

After surgery, the patient was proposed to receive radiotherapy or even imatinib mesylate (800 mg/daily), but eventually no adjuvant therapy was administered, due to patient's desire for a "watchful waiting" tactic. Postoperative follow-up is satisfactory to date and 18 months later the patient remains well, without any signs of tumor recurrence.

## Discussion

DFSP accounts for less than 0.1% of all malignancies and approximately 1% of all soft tissue sarcomas. DFSP is a locally aggressive tumor with a high recurrence rate. Most recurrences of DFSP are detected within 3 years of primary excision. Although metastasis of DFSP is rare (approximately 1-4%), almost all metastatic cases have been associated with local recurrence and a poor prognosis. Most of the patients with metastatic DFSP have died within 2 years. The relative 5-year survival rate for DFSP is 99.2% [1,2].

Among patients, the female to male ratio is 1:1 and the lesions occur most frequently between the second to fifth decades of life. Rarely, DFSP has been reported in newborns and elderly individuals (~80 years). It most commonly appears on the trunk (42-72%), followed by the proximal extremities (16-30%). DFSP rarely occurs above the neck (10-16%) and it is extremely uncommon on the breast [4,5].

Clinically, these neoplasms usually present as a raised, indurated, asymptomatic plaque that may be any combination of blue, red, and brown or flesh colored. The differential diagnosis should always include delayed hypertrophic scar formation, keloids, a recurrent dermatofibroma, or possibly the cutaneous manifestation of underlying 'spindle cell' breast diseases, including a spectrum of tumours with considerable histological similarities, which often require immunohistochemical or even ultrastructural study for accurate identification (fibromatosis, myofibroblastoma, metaplastic carcinoma etc). Chest radiography may be ordered for baseline screening for pulmonary metastasis in high-risk cases, such as recurrence or suspicion for a fibrosarcoma variant of DFSP [2,3,6].

Histologically, the nodular form of DFSP is characterized by a proliferation of plump spindled cells arranged in a monotonous storiform pattern. The cells have little nuclear pleomorphism, and secondary elements such as giant cells, siderophages and chronic inflammatory cells are infrequent. The plaque form of DFSP, however, may show little extension into the subcutaneous tissue and mainly contains slender tumor cells with large, spindle-shaped nuclei, embedded fairly uniformly in the collagen stroma, parallel to the skin surface, while the mitotic figures are sparse. The degree of cellular atypia is higher in nodular lesions than in plaque lesions. Occasionally, DFSP may show focal fibrosarcomatous changes with a characteristic herringbone pattern. The cellular atypia is then even more prominent with hyperchromatic nuclei and more mitotic figures. Histologic subtype, high mitotic index, cellularity, size, location on the head and neck, and recurrent lesions are factors reportedly associated with higher recurrence rates [5,7].

Immunohistochemical studies have shown moderate-to-strong staining of human progenitor cell antigen CD34 in tumor cells. CD34 is a useful marker that allows differentiation of DFSP tumor cells from normal stroma cells and dermatofibroma (DF). In DF, tumor cells are positive for factor XIIIa and are rarely positive for CD34. Additionally, immunostaining using CD34 as a marker is helpful in identifying tumor cells at the surgical margins, particularly when treating recurrent DFSP, in which tumor cell fascicles are often interspersed with the scar tissue. Although CD34 and Factor XIIIa can differentiate most cases of DFSP and DF, the overlap of CD34 and Factor XIIIa expression in both lesions indicates the need to identify other potential immunohistochemical markers [7].

Recently, Stromelysin-3 (ST3) was found to be a useful marker for the differential diagnosis of DF and DFSP. ST3 is a member of the matrix metalloproteinase (MMP) family, which is believed to play a role in tissue remodeling during various processes, such as wound healing and tumour invasion. ST3 is expressed in the majority of cases of DF, whereas DFSPs are only rarely ST3 positive. Tenascin is an extracellular matrix glycoprotein expressed in fibroblasts and the extracellular matrix during embryogenesis and growth. Several studies have shown that there is increased expression of tenascin at the dermal-epidermal junction overlying the spindle cell proliferation in DF, but not in DFSP. Moreover, there is an increasing body of evidence suggesting that DFSP arises from mutated stem cells and demonstrate diffuse strong positivity for the neuroepithelial stem cell protein, nestin. Strong immunoreactivity for nestin is found in DFSP, whereas all DF cases are nestin-negative [8-10].

Surgical excision remains the cornerstone of treatment for DFSP. Complete surgical resection is accepted as the optimal treatment for primary or recurrent DFSP. Most authorities would suggest a margin of 2-3 cm of normal tissue from the gross tumor boundary, with a three-dimensional resection that includes skin, subcutaneous tissue, and the underlying fascia. Despite optimal surgical management, local and regional recurrences are detected in up to 17% of patients with classic DFSP. When surgical margins are inadequate or conservative, recurrence rates increase [5,11].

Radiotherapy provides a useful adjunct, where adequate margins cannot be easily obtained. Radiation can be considered as a possible adjuvant to surgery i) if margins are positive or close after maximal resection, ii) if a large lesion has been excised with negative margins, iii) if there is concern about the adequacy of negative margins, iv) if a recurrent lesion has been resected, or v) if the achievement of wide margins would result in a functional or cosmetic defect. The complete radiotherapy dose ranges from 60-70 Gy [5,11,12].

Nowadays, Mohs micrographic surgery (MMS) has been advocated by several authors as an alternative approach to the use of wide resection surgery with tumor-free margins. Mohs surgical technique allows an immediate microscopic examination of the margins. The process is repeated several times, until a clear margin is achieved. Proponents of this surgical intervention suggest it allows removal of asymmetrical tissue, thus enhancing cosmetic result. In addition, a lower recurrence rate has also been cited by those who recommend MMS as the treatment of choice. However, larger studies and longer follow-up will be necessary to confirm these findings [5,11,13].

Most cases of DFSP feature a specific translocation of chromosomes 17 and 22, which results in constitutive production of platelet-derived growth factor B chain (PDGFB) and stimulation of DFSP growth. This chromosome translocation t (17, 22) is detected in more than 90% of DFSP tumors. The collagen type I alpha 1-platelet-derived growth factor beta (COL1A1-PDGFB) fusion is present in all histological subtypes of DFSP, but not all cases express the fusion transcript. Till now, no association was observed between different COL1A1 breakpoints and clinicopathological parameters. Imatinib mesylate is a potent, selective inhibitor of PDGFR alpha (PDGFRa), PDGFR beta (PDGFRb), BCR-abl, KIT, ARG and c-FMS protein-tyrosine kinases [12,14]. In preclinical studies, imatinib inhibited the growth of DFSP cells, as well as fibroblasts transformed by the t (17; 22) chromosomal rearrangement both in vitro and in vivo. On October 19, 2006, the US Food and Drug Administration (FDA) granted approval for imatinib, as a single agent for the treatment of adult patients with unresectable, recurrent, and/or metastatic DFSP. With limited clinical data to date, a response rate of approximately 65% has been achieved among DFSP patients treated with imatinib (recommended dose 800 mg/d). A small subset of DFSP lacking the classic t (17, 22) gene aberration seems to have no response to imatinib. Thus, cytogenetic studies that confirm PDGFB gene rearrangement may be necessary in predicting future clinical response, prior to imatinib therapy administration [5,11,15,16].

## Competing interests

The authors declare that they have no competing interests.

## Authors' contributions

DMD was responsible for original conception and design, editing, English editing, search of the literature, correction, editorship of the manuscript. LAKK was responsible for acquisition, analysis and interpretation of data, English editing and search of the literature. IKA was responsible for the histology consulting and pathology examination. APT was responsible for correction, editing, revision, and approval of the final version.

## Consent

Written informed consent was obtained from the patient for publication of this case report and accompanying images. A copy of the written consent is available for review by the Editor-in-Chief of this journal.
